# Influence of Al Alloying on the Electrochemical Behavior of Zn Electrodes for Zn–Air Batteries With Neutral Sodium Chloride Electrolyte

**DOI:** 10.3389/fchem.2019.00800

**Published:** 2019-11-20

**Authors:** Yasin Emre Durmus, Saul Said Montiel Guerrero, Hermann Tempel, Florian Hausen, Hans Kungl, Rüdiger-A. Eichel

**Affiliations:** ^1^Institute of Energy and Climate Research–Fundamental Electrochemistry (IEK-9), Forschungszentrum Jülich GmbH, Jülich, Germany; ^2^Department of Electrical Engineering and Information Technology, University of Duisburg-Essen, Duisburg, Germany; ^3^IESW, Institute of Physical Chemistry, RWTH Aachen University, Aachen, Germany

**Keywords:** metal–air battery, zinc/aluminum alloy, neutral aqueous electrolyte, corrosion, aluminum activation

## Abstract

Zn alloy electrodes containing 10 wt. % Al were prepared to examine the applicability as anodes in primary Zn–air batteries with neutral 2M NaCl electrolyte. These electrodes were investigated by electrochemical measurements and microscopic techniques (SEM, LSM, AFM). Based on the cyclic voltammetry and intermediate term (24 h) discharge experiments, the only active element in the as-prepared alloy was found to be Zn. It was further confirmed by LSM that Zn rich areas dissolved while Al remained passive during discharge. The passive state of Al was also demonstrated by conductive AFM investigations on the as-cast alloy surfaces. The results on potentiodynamic polarization and weight loss measurements indicated that the alloy electrode was less prone to corrosion than pure Zn electrode. The electrochemical behavior of the electrodes was modified under certain cathodic polarization previous to measurements. Accordingly, originating from Al activation due to application of cathodic potentials, potentiodynamic polarization studies showed a clear shift on the corrosion potentials of the alloy toward more negative values. On the basis of these results, with the precondition of Al activation prior to discharge experiments, the effect of Al alloying on the Zn electrodes was revealed as temporarily enhanced potentials on the discharge profiles in comparison to pure Zn electrodes.

## Introduction

Metal–air batteries, among other emerging technologies, provide a very promising electrochemical energy storage technology due to possessing high theoretical energy densities while using low cost, safe, and abundant electrode materials. Specifically when referring to Zn, Al, Mg, Si, and Fe, the availability and price of the materials in terms of production make such battery systems highly resource efficient (Wedepohl, 1995; Gelman et al., 2016; US Geological Survey, 2018; Weinrich et al., 2019). Among the aqueous metal–air systems, Zn–air is the most advanced as the primary alkaline Zn–air batteries, which have been under development for quite a long time (Blurton and Sammels, 1979; Linden and Reddy, 2002), are already commercially available for broad low-power demanding applications (e.g., hearing aids, warning lights, remote signals). With respect to secondary rechargeable batteries, Zn–air systems suffer from various technical challenges and thus, they are still under intensive research and development (Rahman et al., 2013; Li and Dai, 2014; Pei et al., 2014; Mainar et al., 2018; Yi et al., 2018; Liu et al., 2019).

Despite its high theoretical specific energy (1,352 Wh/kg_Zn_), primary alkaline Zn–air batteries could achieve only up to 700 Wh/kg_Zn_ while secondary alkaline Zn–air batteries are limited to 300–500 Wh/kg_Zn_ (Li et al., 2013; Li and Dai, 2014). The technical challenges mainly originate from (i) shape change, stability, and cyclability of Zn anode (Arlt et al., 2014; Turney et al., 2017; Lee et al., 2019), (ii) inefficient bifunctional catalysts (Cheng and Chen, 2012; Wang et al., 2014; Niu et al., 2018), (iii) stability of carbon-based air cathode (Cheng and Chen, 2012; Wang et al., 2014; Li and Lu, 2017), and (iv) impurities in ambient air composition (e.g., CO_2_) (Ko and Juang, 1983; Drillet et al., 2001; Schröder et al., 2015). These aforementioned challenges are mostly specific for alkaline Zn–air batteries; employing the conventional alkaline electrolyte is highly attractive as it offers superior ionic conductivities (Gilliam et al., 2007). Some of these problems such as dendrite formation upon cycling, corrosion of carbon-based air cathode and carbonate formation in electrolyte (when in contact with CO_2_ from ambient air) can mostly be avoided by using aqueous neutral electrolytes (Jindra et al., 1973; Mainar et al., 2018). Reduced solubility of zinc, low concentration of OH^−^, and limited CO_2_ capture from ambient air would result in improved performance of Zn–air in terms of prolonged and stable cyclability.

The first application of chloride-based neutral aqueous electrolyte in Zn–air batteries dates back to 1973, when Jindra et al. (1973) reported a primary Zn–air cell with 5M NH_4_Cl aqueous neutral electrolyte. Galvanostatic discharge experiments showed that the battery could be operated at 0.9–0.95 V with 50 mA (10 mA/cm^2^ of cathode) while corrosion rates of Zn being at least one order of magnitude lower than in alkaline electrolytes (Jindra et al., 1973). More importantly, carbonate formation by absorption of CO_2_ from ambient air was not observed. The first electrically rechargeable Zn–air battery was reported only recently by Amendola et al. (2012). The Zn–air battery employing ZnCl_2_ + NH_4_Cl electrolyte was demonstrated with operating voltages between 0.9 and 2.1 V during cycling. In 2014, Goh et al. studied the effects of salt concentration, pH, and electrolyte additives (polyethylene glycol (PEG) and thiourea) on the zinc electrodeposition in NH_4_Cl based electrolyte (Goh et al., 2014). Accordingly, the Zn–air battery with the optimum composition of the electrolyte could be cycled for 115 times over 1,400 h. An even better cycling stability with 540 cycles over 2,100 h was achieved by Sumboja et al. who reported a Zn–air battery providing 1 and 2 V at 1 mA/cm^2^ on discharge and charge; respectively (Sumboja et al., 2016). Although the ORR and OER activities of MnO_x_ catalysts were lowered in neutral electrolyte, the battery could maintain extended cycling performance due to the reduced carbon corrosion and carbonate formation in the electrolyte. Basing on these results, a more recent study by Clark et al. reported a continuum model to simulate the performance of Zn–air batteries with ZnCl_2_ + NH_4_Cl electrolyte (Clark et al., 2017). Using the model, the influence of the concentration and pH gradients in the electrolyte during battery operation was determined. Moreover, the composition of the electrolyte and the cell design were optimized considering factors such as pH stability, final discharge products, and energy density. Accordingly, the optimum composition of the electrolyte was reported as 0.5M ZnCl_2_ + 2M NH_4_Cl at pH 7.

In the previous studies on Zn–air batteries with neutral aqueous electrolyte, the investigations were mostly focused on the electrolyte composition of NH_4_Cl based solutions and the activity of the electrocatalysts while using Zn plate or Zn sheet as an anode material. The influence of the Zn anode composition as well as other chloride-based neutral aqueous solutions on the performance of Zn–air batteries are other important issues. More specifically, alloying of Zn with other metals such as Al might be an interesting option as an anode material to improve the electrode potential and corrosion behavior. Combination of two metals in an electrode may influence the potential and corrosion currents according to the mixed potential theory (Wagner and Traud, 1938); hence, possibly resulting in a better electrode performance with higher discharge energy.

The main objective of the present paper is to investigate the influence of Al alloying on the electrochemical behavior of Zn electrodes in neutral 2M NaCl electrolyte. Zn-Al alloy electrodes containing 10 wt.% Al were prepared and microstructures were evaluated with scanning electron microscopy. The electrochemical activities of alloy, pure Zn and pure Al electrodes were investigated in cyclic voltammograms. The intermediate term (24 h) discharge (or stripping/dissolution due to three-electrode setup) of the three electrodes were characterized under current densities of 0.1, 0.25, 0.5, and 1 mA/cm^2^. Subsequent to the discharge, the alloy surface was subject to laser scanning microscopy (LSM) analysis in order to obtain better insights into dissolution of the anode components. The surface of the as-cast alloy was further analyzed by atomic force microscopy (AFM) to investigate the local conductivities of alloy constituents. The corrosion parameters such as corrosion potentials and corrosion currents were calculated from potentiodynamic polarization curves while gravimetric corrosion rates were obtained from weight loss experiments. Potentiodynamic polarization studies showed a clear shift on the corrosion potential of the alloy toward more negative values due to Al activation originating from cathodic potentials. Basing on these results, a cathodic potential pulse was applied to the electrodes prior to discharge. Accordingly, the effect of Al alloying on the Zn electrodes was revealed as temporarily enhanced potentials on the discharge profiles. Overall, the effect of Al alloying resulted in as “temporarily” enhanced discharge potentials and reduced corrosion rates in comparison to pure Zn electrodes in neutral 2M NaCl electrolytes.

## Experimental Section

### Materials Preparation and Chemicals

Zinc-Aluminum alloy (Zn-10Al) was prepared from cold drawn tempered zinc rods (4 N, Alfa Aesar) and aluminum rods (Puratronic grade, 5 N, Alfa Aesar) with a composition of 90 wt.% Zn and 10 wt.% Al. The pure Zn and Al electrodes were prepared directly from the rods. The materials were melted in a tube furnace (Gero REST-E 400/6, Germany) at 700°C for 3 h and subsequently cooled down to room temperature under continuous flow of argon gas. Composition analysis of the alloy was obtained by inductively coupled plasma optical emission spectrometry (ICP-OES) as shown in Table 1.

**Table 1:** ICP-OES analysis of the as-cast Zn-10Al alloy.

Alloy	Si/wt.%	Mg/wt.%	Fe/wt.%	Pb/wt.%	Cu/wt.%	Al/wt.%	Zn/wt.%
Zn-10Al	<0.001	<0.001	0.001	<0.005	0.001	9.93	Balance

The samples were cut into discs, the pieces were embedded in cold mount epoxy (EpoFix, Struers) and the surfaces were ground with 800 SiC paper. The corresponding areas for the Zn, Zn-10Al alloy and Al electrodes are 1.32, 1.32, and 0.77 cm^2^, respectively.

2M NaCl solutions were prepared by dissolving NaCl crystals (≥99.5%, Merck-Millipore) in deionized water (conductivity <0.1 μS/cm, PURELAB Elga). The solutions were degassed by flowing argon for several minutes (>10 min) in order to remove dissolved oxygen. Adjustment of the pH was done by adding small amounts of 0.1M NaOH solution until reaching pH 7. The pH of the NaCl solutions was measured by a pH meter (Duo S213, Mettler Toledo).

### Electrochemical Methods and Corrosion

A three electrode setup with Zn, Al, and Zn-10Al as working, Pt mesh as counter, and silver/silver chloride (Ag/AgCl) as reference electrodes was employed in the experiments. The cell volume was 20 mL. Electrochemical cyclic voltammetry, galvanostatic, and potentiodynamic polarization experiments were carried out with a Biologic VMP3 potentiostat. Cyclic voltammetry (CV) and potentiodynamic polarization were conducted with a scan rate of 5 mV/s. Galvanostatic discharge was performed by applying 0.005, 0.01, 0.05, 0.1, 0.25, 0.5, and 1 mA/cm^2^ discharge current densities. For the Al activation experiments, a potentiostatic cathodic pulse at −1.6 V vs. Ag/AgCl was applied for 60 min prior to discharge. All the experiments were carried out in a climate chamber (Binder KMF115) to ensure constant controlled ambient conditions.

The gravimetric corrosion experiments were measured over 7 days in 2M NaCl with pH 7. After exposure, the samples were cleaned with saturated glycine solution according to ISO 8407 standard (ISO 8407, 2009) and the weight losses were determined by using an analytical balance with an accuracy of 0.01 mg (XA205, Mettler Toledo).

### Microstructure and Elemental Analysis

The previously embedded samples were ground and polished using a Tegramin system (Struers, Germany) for microstructural analysis. The final polishing steps consisted of 3 μm (DiaPro Mol B) and 1 μm (DiaPro Nap B) water–based diamond suspensions with lubricating liquid. The microstructures were characterized by a confocal laser scanning microscope (OLS4100, Olympus Corp., Japan) and by scanning electron microscopy (Quanta 650, FEI, USA) using the concentric backscatter electron (CBS) detector and energy-dispersive X-ray spectroscopy (EDS) (Octane Super Detector, EDAX, USA). Applied acceleration voltages in the SEM measurements were 20 kV.

Atomic force microscopy (AFM) images of the polished samples have been obtained with a Bruker Dimension ICON (St. Barbara, CA, USA) inside a glove box (O_2_ < 1 ppm, H_2_O < 1 ppm) in conductive-AFM mode. As cantilevers SCM-PIT-V2 (Bruker, St. Barbara, CA, USA) with a Platinum-Iridium coating and a nominal spring constant of 3 N/m as well as a tip radius of 25 nm have been used as received and calibrated individually. For conductivity experiments, the sample was contacted from underneath.

Inductively coupled plasma optical emission spectrometry (ICP-OES) was conducted with ThermoFischer scientific iCAP 7600 spectrometer with Echelle-optic and CID-semiconductor detector. For the analysis, 50 mg of alloy sample was dissolved in 3 mL HNO_3_/3 mL HCl solution with a total volume of 50 mL. Two independent parallel digestion solutions of this sample were analyzed by ICP-OES.

## Results and Discussion

### As-Cast Microstructure of the Alloy

**Figure 1:**
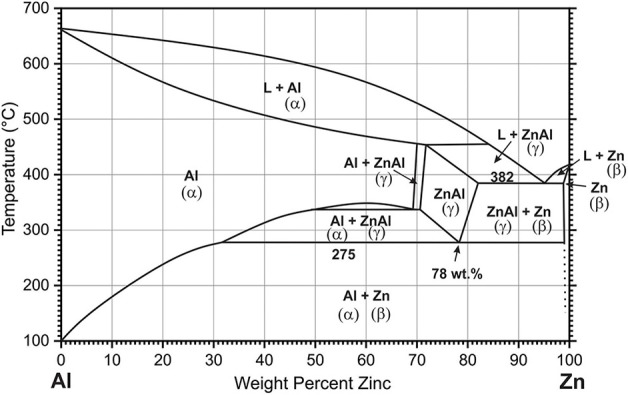
Phase diagram of binary Zn-Al alloy. The figure is constructed from Mondolfo (1976), Goldak and Gordon Parr (1964), and Wei et al. (2019).

The microstructure of the Zn-10Al alloy undergoes several transformations when cooled down from the melt. According to the phase diagram (Figure 1) for the Zn-10Al alloy the liquidus is at ~410°C at which primary γ-ZnAl starts to nucleate from the melt. γ-ZnAl denotes an fcc phase containing 70–76 wt.% Zn with possibly ordered structure (Presnyakov et al., 1961; Goldak and Gordon Parr, 1964; Mondolfo, 1971). Under equilibrium conditions solidification is completed at the eutectic temperature (382°C), where the alloy consists of primary γ-ZnAl and eutectic from β-Zn and γ-ZnAl. On further cooling to 275°C, the material undergoes an eutectoid transformation, in which—depending on the cooling conditions—α-Al and β-Zn form from the γ-ZnAl. While the equilibrium phases of α-Al and β-Zn are quite low in content of the respective other component, microstructure constituents with deviating composition might be remaining under non-equilibrium conditions.

**Figure 2:**
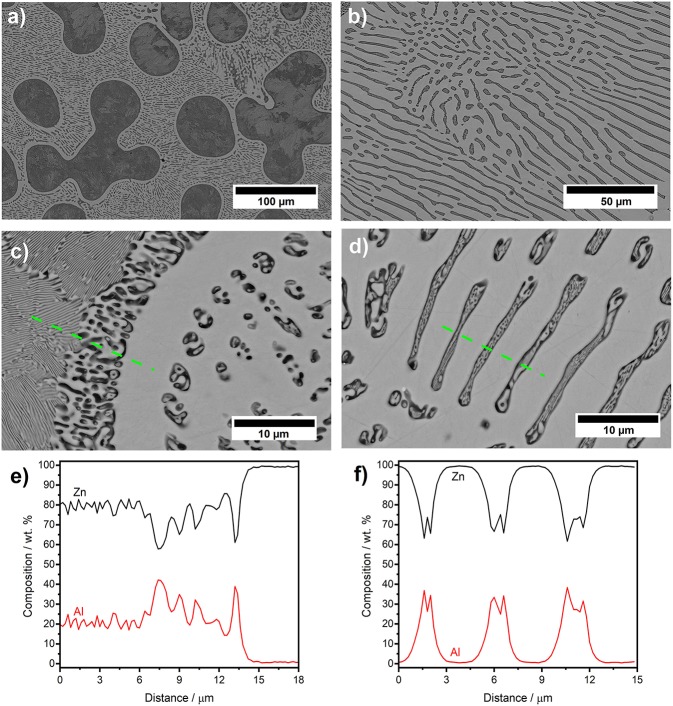
SEM images recorded with backscatter electron detector of as-cast Zn-10Al alloy, **(a)** 500× magnification overview of microstructure constituents, **(b)** 1,100× magnification of the lamellas and islands, **(c)** 5,000× magnification of the boundary region between the dark and bright zone, **(d)** 5,000× magnification of the lamellas and islands, **(e,f)** are the results from EDS analysis for line scans indicated in **(c,d)**, respectively.

At low magnification (Figure 2a), the SEM image of the alloy shows large dark areas, small dark lamellas, and islands surrounded by light areas covering the rest of the surface. The light areas are the β-Zn phase. The shape and the size of the dark areas varies with typical size of up to 50 μm. They are surrounded by Zn-rich halos with a width of <10 μm, characteristic for hypereutectic Zn-Al alloys (>5 wt.% Al) at low cooling rates (Bluni et al., 1995; Yang et al., 2017).

At higher magnifications more distinct features in the region toward a 2–5 μm wide rim of the large dark areas as well as in their inner part (Figure 2c) can be identified. In the near rim regions, the material splits into two phases on a 100–500 nm scale, with one of them forming round shaped grains (Wei et al., 2019). Lamellar structures, with spacings in the range from 50 to 100 nm can be distinguished in some regions of the inner part of the large dark areas (Zhu, 2004). The nanostructures are attributed to formation of the eutectoid (Zhu, 2004; Wei et al., 2019). While for the large area bright regions the EDS detects a Zn-content of almost 100 wt.%, the eutectoid in the inner part contains 80 wt.% Zn and 20 wt.% Al on average (Figures 2c,e). Resolution within these nanostructures of the eutectoid is not possible due to the limitation of the EDS spot size. The somewhat larger structures of the eutectoid in the rim area allow for a better resolution indicating the Al content in the dark areas thereof is higher.

The dark regions appearing on low magnification as lamellae (Figure 2b) and islands are originally formed during the eutectic reaction. In the regions with predominantly lamellar structures the interlamellar spacing λ is 3 μm approximately. However these structures are not perfectly laminar, but contain substantial rod shaped constituents appearing as islands, which is typical for Zn-Al alloys with Al contents higher than 7 wt.% (Bluni et al., 1995). Therefore, although the value for lamellar interspacing roughly matches the results for this type of alloys at relatively low cooling rates, it cannot be considered a precise result for establishing correlations (Bluni et al., 1995). SEM images at higher magnifications reveal, that within both—lamellae and islands—a nanostructured framework is formed (Figure 2d), which most likely corresponds to α-Al/β-Zn- eutectoid. For the average content of the eutectoid lamellae regions the EDS indicates 70 wt.% Zn and 30 wt.% Al (Figures 2d,f).

### Electrochemical Behavior of the Alloy

#### Cyclic Voltammetry

**Figure 3:**
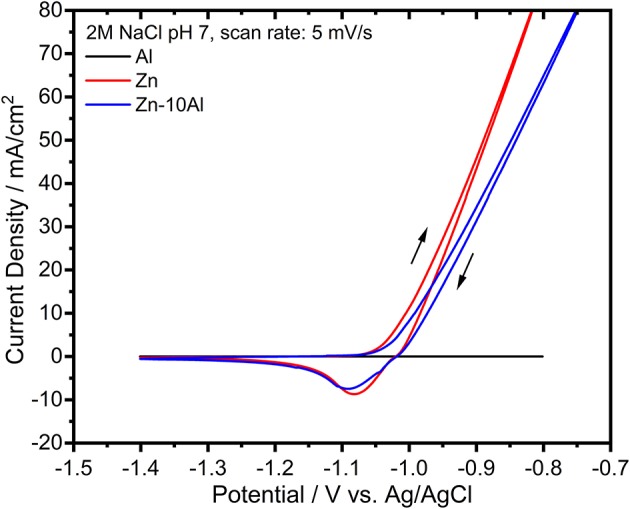
Cyclic voltammograms of pure Al and Zn, and Zn-10Al alloy electrodes in 2M NaCl at pH 7. The scan was initiated toward anodic potentials as shown with the arrows. The scan rate was 5 mV/s.

The electrochemical activities of the prepared alloy, pure Al, and pure Zn electrodes were investigated with cyclic voltammetry (CV) experiments. Figure 3 demonstrates the CVs of Al, Zn, and Zn-10Al alloy within a potential range of −1.4 to −0.8 V vs. Ag/AgCl in 2M NaCl with pH 7. The voltammogram for Al does not show any significant electrochemical activity during both forward and reverse scans. The Zn electrode, on the other hand, shows onset of oxidation starting at −1.08 V vs. Ag/AgCl. The reverse scan reveals a reduction peak for Zn around −1.08 V vs. Ag/AgCl. Concomitant to Zn reduction, originating from the nature of the aqueous electrolyte, there is also a hydrogen evolution at such relatively high potentials; however, at a much slower rate in comparison to the Zn reduction/deposition reaction. The CV for the Zn-10Al alloy is almost identical to pure Zn.

According to the CV experiments, alloying of Zn with Al does not impede any pronounced influence on the electrochemical behavior. Considering the similarity of CVs of Zn and Zn-10Al in combination with the result that Al does not have any electrochemical activity, Zn is suggested to be the only active component in the alloy with neutral electrolyte. Aluminum, for which the theoretical electrochemical potential is more negative, is present as passivated in the pure Al as well as in the alloy electrodes.

It is also noteworthy to mention that both Zn and Zn-10Al electrodes exhibit a steady increase of the oxidation current during the anodic scans without any visible oxidation peak over the complete anodic potential range. Generally, typical cyclic voltammograms show a single or multiple oxidation peaks after which the currents start to decrease due to depletion of active species or formation of passivation layer. Remaining within the electrochemical stability window of the electrolyte, a continuous increase of the oxidation current as shown in Figure 3 indicates that the oxidation reaction of Zn continues at relatively high anodic overpotentials via pitting mechanism. Pitting is known as localized accelerated dissolution of a metal as a result of localized breakdown of passive film (Zhang, 1996; Frankel, 1998). The presence of aggressive anionic species, such as chloride (Cl^−^), is mainly the cause for pitting mechanism (Zhang, 1996; Frankel, 1998; Miao et al., 2007).

#### Galvanostatic Discharge

**Figure 4:**
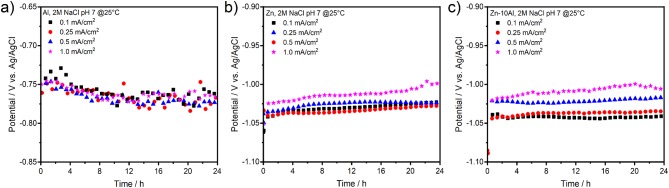
Galvanostatic discharge (or stripping) experiments of **(a)** pure Al, **(b)** pure Zn, and **(c)** Zn-10Al alloy electrodes. The discharge current densities were 0.1, 0.25, 0.5, and 1.0 mA/cm^2^ over 24 h.

Figure 4 presents the galvanostatic discharge experiments (or stripping experiments due to half-cell setup) of Al, Zn, and Zn-10Al alloy in 2M NaCl with pH 7 under various current densities. To examine the possible influence of Al alloying on the electrochemistry and to prevent significant pitting formations, current densities only up to 1 mA/cm^2^ were employed.

Discharging the Al electrode in the neutral electrolyte results in relatively low anodic potentials. As shown in Figure 4a, even at very low current densities (0.1 mA/cm^2^), the Al could anodically be utilized only at about −0.76 V vs. Ag/AgCl. In case of Zn and Zn-10Al, both electrodes provided discharge potentials around −1.03 V vs. Ag/AgCl. There was no pronounced difference on the discharge potentials for current densities up to 1 mA/cm^2^ and the potentials of the three electrodes were stable over 24 h. Overall, the alloy showed very similar discharge profiles to Zn electrode during the galvanostatic studies.

The elemental steps of the reactions governing the dissolution of Zn and Al upon discharge are as follows:

(1) $$\begin{array}{r} Dissolution\;of\;zinc:Zn_{\left(s\right)}\to \;Zn_{\left(aq\right)}^{2+}+{2e}^{-} \end{array}$$

(2) $$\begin{array}{r} Dissolution\;of\;aluminum:Al_{\left(s\right)}\to \;Al_{\left(aq\right)}^{3+}+{3e}^{-} \end{array}$$

The formed aqueous ions, Zn^2+^ and Al^3+^, would further complex with other species in the electrolyte. In the presence of NaCl, when the solubility limits are exceeded, zinc hydroxide chloride, or simonkolleite [Zn_5_(OH)_8_Cl_2_·H_2_O], as well as zinc oxide (ZnO), can be formed in case of zinc (Qu et al., 2005; Mouanga et al., 2010), while for Al, aluminum hydroxide [Al(OH)_3_], aluminum oxide (Al_2_O_3_) and even aluminum chloride (AlCl_3_) species may be formed (Foley and Nguyen, 1982; Vargel, 2004). Moreover, if both metals are present on the electrode surface, as for Zn-10Al, layered double zinc-aluminum hydroxides can also be produced in neutral and near neutral aqueous solutions (Vu et al., 2013; Salgueiro Azevedo et al., 2015). The existence of the end products are highly dependent on the solution pH (Miao et al., 2007; Vu et al., 2013).

According to the Pourbaix diagrams, Al and Zn are thermodynamically not stable in aqueous solutions over the complete pH range since their immunity regions are below the stability window of water (Pourbaix, 1974). Thereby, these metals react with aqueous electrolyte instantly to produce stable species which are in the form of dissolved ions (corrosion region) or solid oxide/hydroxides (passivated region). In the pH range of interest for our study (pH 7) Al remains in the “passivated region” while Zn is in the “corrosion region.”

Under these conditions, galvanostatic discharge of Al through the passive layer is only possible if the passive layer is dissolved or attacked by the adsorbed Cl^−^ ions to initiate pits. Once the Al is exposed to electrolyte, discharge (active dissolution) continues via pitting at a stable potential at least over 24 h as shown in Figure 4a. Dissolution kinetics and the conductivity of electrolyte are high enough that resulted in similar steady state potentials for 0.1 and 1 mA/cm^2^ current densities. Smolijko et al. also reported that even under current density of 20 mA/cm^2^, Al electrodes could provide discharge potentials around −0.75 V vs. Ag/AgCl in 2M NaCl electrolyte (Smoljko et al., 2012). There was a linear increase on the steady state potentials for anodic currents between 20 and 100 mA/cm^2^; the discharge potential under 100 mA/cm^2^ was around −0.6 V vs. Ag/AgCl (Smoljko et al., 2012). Note that such high current densities may result in severe pit formations which could be detrimental for the stability of the discharge experiments in a battery application.

The Zn and Zn-10Al electrodes exhibit discharge potentials of about 0.28 V more negative than that of Al under the same current densities. Due to the fact that Zn is in the corrosion regime, there is no compact passive layer on the surface. Consequently, the electrochemical reactions on the Zn and alloy electrodes occur at much lower electrode polarizations; thus, more negative steady state potentials are obtained as shown in Figures 4b,c. Almost identical discharge profiles of Zn and Zn-10Al alloy electrodes support the CV results that Al does not contribute or influence the electrochemistry of the electrode and Zn is the only active component. Since the discharge potential is not close to Al discharge (or pitting) potential (see Figure 4a), the Al in the alloy remains passive.

### Surface Analysis of the Alloy

#### LSM

**Figure 5:**
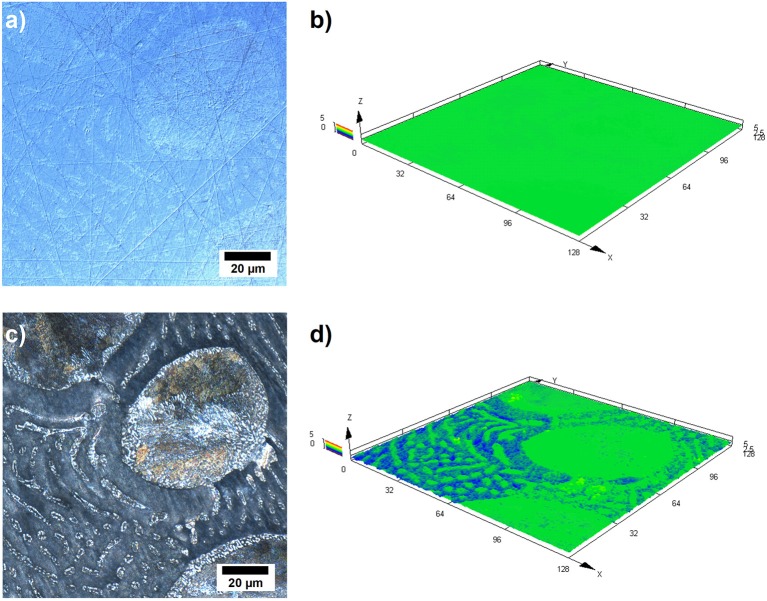
Laser scanning micrographs of Zn-10Al alloy electrodes: **(a)** 2D image of the surface prior to galvanostatic discharge, **(b)** 3D image of the same area shown in **(a)**, **(c)** 2D image of the same area after 3 h of galvanostatic discharge with 1 mA/cm^2^ in 2M NaCl electrolyte with pH 7, **(d)** 3D image of the same area shown in **(c)**.

Further investigations have been conducted by laser scanning microscopy (LSM) in order to obtain better insight into the dissolution behavior of the alloy during the galvanostatic discharge experiments. Figure 5 illustrates the LSM images of the Zn-10Al alloy which was discharged with 1 mA/cm^2^ current density for 3 h. Figures 5a,b represent an area on the surface prior to the experiment while Figures 5c,d represent the same area subsequent to 3 h of discharge.

From the comparison of the Figures 5a,c, it can clearly be seen that the contrast difference between the microstructures are enhanced upon discharge. The larger grains containing eutectoid Zn-Al became more visible. Also the γ-phase lamellas and islands in the eutectic can be distinguished. Moreover, the β phase (Zn) which fills the areas between the other phases seems partially etched away. A comparison of the height profiles is shown by the Figures 5b,d where a 3D image of the same area is provided. According to Figure 5d, there is a height difference of almost 3 μm between the Zn (β phase) and the other phases which contain some Al.

The LSM images support the previous findings that the active component in the alloy is Zn since the pure Zn phase is dissolved from the alloy surface as shown in Figure 5. Otherwise, a more homogeneous dissolution would be expected on the surface. However, basing only on the LSM images, it is difficult to state that the other phases remain completely inactive because a certain fraction of Zn is present also in the Al containing constituents. It could be the case that Zn from the other phases might also be dissolving but at a much slower rate.

#### AFM

The surface of the prepared Zn-10Al alloy electrode was further investigated by conductive atomic force microscopy (c-AFM). While LSM is not sensitive to chemical or mechanical differences of the surfaces under investigation, c-AFM is measuring electrical properties simultaneously next to topographical features as well as the lateral force.

**Figure 6:**
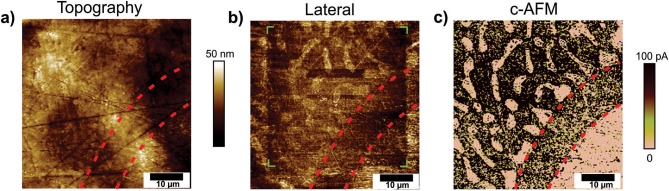
Atomic force microscopy images of as-cast Zn-10Al alloy electrodes. **(a)** The surface topography, **(b)** Lateral force, **(c)** conductive-AFM (c-AFM). All the images were recorded simultaneously. In **(b)**, the green highlighted edges of the quadratic area indicate the previous scan. In all the images, as a guide to the eye dashed lines are shown corresponding to part of the halo structure separating the large Al-rich grain from the eutectic lamellas and islands.

The surface topography of as-cast Zn-10Al alloy is depicted in Figure 6a. The morphology shows several bumps and depressions on an overall rather smooth surface. Additionally, it exhibits several scratches in various directions which can be attributed to the polishing process of the surface. The observed surface morphology is similar to the very flat surface in LSM (Figure 5b). The typical microstructural composition of the Zn-10Al alloy as found in SEM experiments (Figure 2) cannot be distinguished from topography. This can be expected since the topography mode does not provide any material-specific contrast and thus, cannot differentiate between Zn and Al rich constituents.

Lateral forces between the AFM tip and the alloy have been probed simultaneously to the surface morphology. Such lateral forces are very sensitive even to subtle materials changes, as expected to be present in the system under study. Figure 6b shows the recorded lateral force image (trace). A quadratic part of lower lateral forces (darker appearance) is recognizable as indicated by the highlighted edges. The size of this structure is 35 × 35 μm^2^, corresponding to the scan size of the beforehand image. Two different materials can be differentiated in the lateral force image: Several regions of higher lateral force (brighter appearance) are apparent in form of isolated areas with a few μm in size especially in the top half of the image as well as in form of a larger sized quadrant in the bottom right corner. These regions are surrounded by a darker matrix. These structures do not have a counterpart in the topography image and can therefore be attributed to different materials, such as β-Zn and Al containing parts of the alloy. A closer inspection reveals that the brighter grains exhibit a similar lateral force inside and outside the square while the surrounding area shows reduced lateral forces inside the quadratic feature. It can be concluded that the surrounding is stronger affected by previous mechanical load than the isolated areas.

Figure 6c demonstrates the local conductivity of the surface area and was recorded simultaneously to the images shown in Figures 6a,b. Here, a very strong material contrast of the surface is observed. Interestingly, these properties are not reflected in the topography image but in the lateral force image and can therefore directly related to β-Zn and Al containing parts in accordance with the previously discussed findings from SEM and LSM. While the isolated Al containing areas possess only marginal conductance (bright color), the β-Zn surrounding (darker appearance) demonstrates a significantly higher conductance. The scratches originated in the polishing process do not contribute to the overall conductivity. As a guide to the eye dashed lines are shown in Figures 6a–c for direct comparison and separate the bottom right Al containing quadrant grain from the smaller isolated Al containing areas embedded in the β-Zn phase. Interestingly, a clear halo feature of β-Zn phase is encompassing the quadrant-like Al containing structure. This halo can also been noticed in the SEM as well as LSM images shown in Figures 2, 5 and is between 7.5 and 10 μm in width, exhibiting a rather conductive region. According to the c-AFM experiment, the β-Zn regions exhibit high conductivity, indicating the metallic character of the surface, whereas a passivating layer which limits the conductance is present on the surface of the Al containing areas.

### Corrosion Behavior of the Alloy

**Figure 7:**
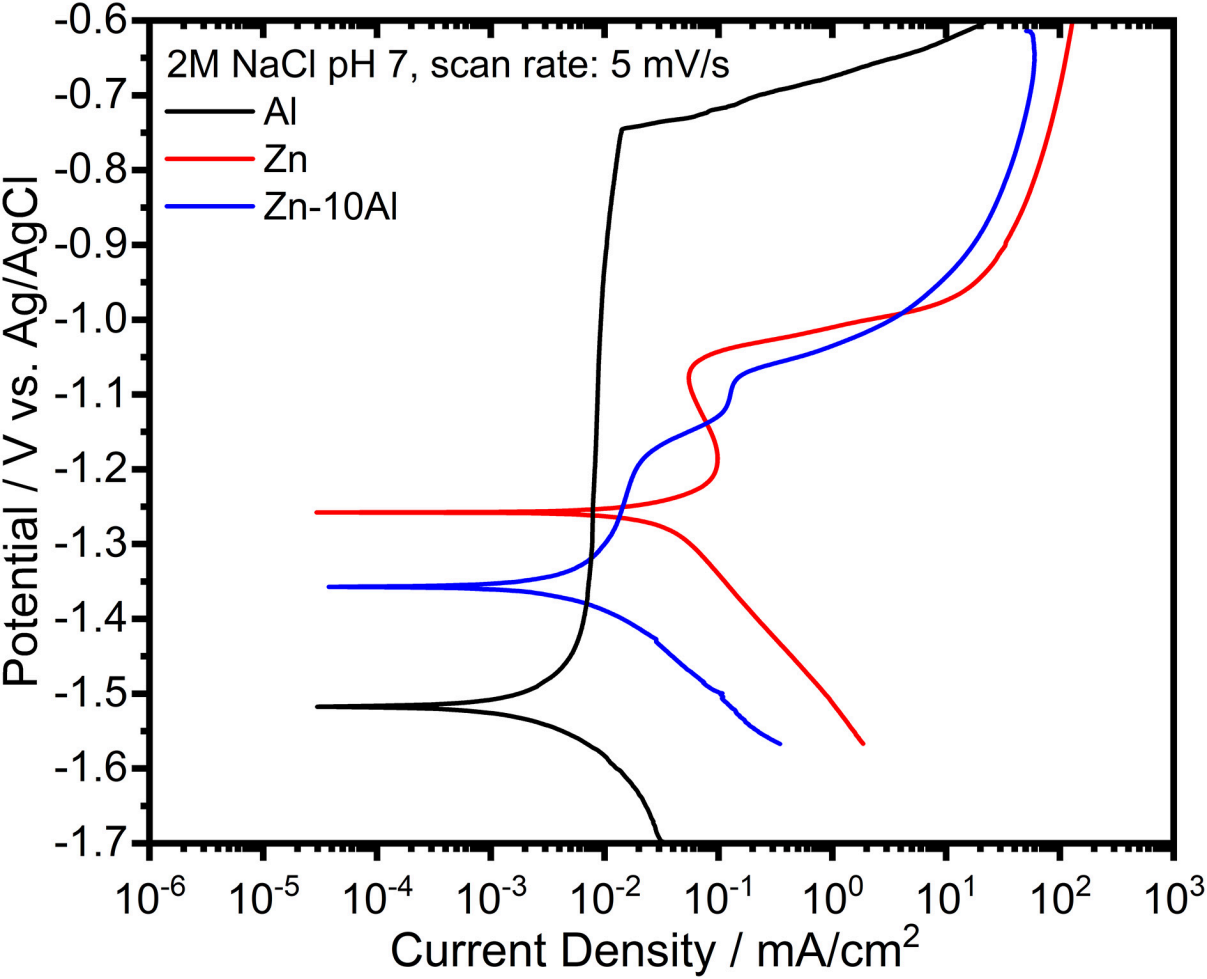
Potentiodynamic polarization curves of pure Al and Zn, and Zn-10Al alloy electrodes in 2M NaCl solutions with pH 7. The scans for each electrode was initiated at cathodic potentials toward the anodic direction with a scan rate of 5 mV/s.

The potentiodynamic polarization curves of pure Al and Zn, and Zn-10Al alloy in 2M NaCl solution are shown in Figure 7. The corrosion potential (or open circuit potential) of Al was about −1.5 V vs. Ag/AgCl; thus being more negative than for the Zn and the alloy while the cathodic and anodic currents were shifted to lower values. The shape of the anodic curve reveals a broad passive region down to −0.76 V vs. Ag/AgCl with a passive current of about 7 μA/cm^2^. The abrupt increase of the current at the end of the passive region is attributed to the breakdown of the passive film followed by an onset of the pitting mechanism (Breslin et al., 1993; El Shayeb et al., 2001; Abedin and El, 2004).

The polarization curve of Zn represents a different behavior from than that of Al. The curve in general was shifted toward higher currents while the corrosion potential of Zn was at more positive value (−1.24 V vs. Ag/AgCl). During the anodic polarization, a peak was observed which represents the anodic oxidation of Zn. The anodic current starts to increase around −1.0 V vs. Ag/AgCl due to initiation of pitting. The Zn-10Al alloy exhibits a polarization curve which differs from pure Zn. The corrosion potential was enhanced toward negative direction by almost 100 mV in comparison to Zn. The anodic scan reveals two peaks that could be assigned for Al and Zn oxidation. Similar to Zn, there is pitting initiation around −1.0 V vs. Ag/AgCl.

The corrosion parameters extracted from the polarization curves are shown in Table 2. The corrosion current densities, which represents the rate of material dissolution, were obtained by Tafel fits in the range of ±50 mV relative to OCP. Among the three investigated electrodes, Zn exhibits the highest corrosion current density (16.2 μA/cm^2^) while Al is the least susceptible electrode to corrosion with 1.5 μA/cm^2^. The effect of Al alloying is visible on the corrosion current density that is lowered by almost 5 times (3.4 μA/cm^2^) in comparison to Zn electrode. The corrosion rates calculated from gravimetric weight loss experiments are summarized in Table 2. The intermediate term stability of the electrodes was investigated in 2M NaCl at pH 7 over 7 days of exposure. The Al electrode did not show any weight loss owing to its passive behavior under steady state conditions. As expected, the highest corrosion rate was found for the Zn while the Zn-10Al alloy revealed more stable behavior in such immersion conditions. The results of the gravimetric weight loss experiments also confirm that Al alloying is advantageous in terms of reduced corrosion rates.

**Table 2:** Corrosion parameters obtained from potentiodynamic polarization curves and gravimetric weight loss experiments.

	**Polarization curves**		**Weight loss**
	**E_***corr***_ (Mv)**	**J_***corr***_ (μA/cm^**2**^)**	**m_***corr***_ [μg/(cm^2^·h)]**
Zn	−1,242	16.2	4.43 ± 0.89
Zn-10Al	−1,350	3.4	2.61 ± 0.92
Al	−1,517	1.5	0

Considering the results from CV and galvanostatic discharge experiments, a different behavior of the alloy was observed in the potentiodynamic polarization curves. The essential difference originates from the fundamentals of the polarization experiment; the scan starts from the cathodic potentials toward anodic direction. During the cathodic range, the current is produced from either dissolved oxygen reduction or water reduction reactions (Zhang, 1996). The solutions were always degassed with Ar prior to experiments; hence, water reduction reaction is more likely to take place. Nevertheless, the common product of both reactions are hydroxyl ions (OH^−^). Continuous cathodic polarization gives a rise to alkalinity at the local spots on the alloy surface due to the generation of OH^−^. Accordingly, local alkalization results in hydration and/or dissolution of aluminum oxide and eventually, revealing the bare Al surface. This phenomenon is known as “cathodic corrosion of Al” or “activation of Al by cathodic polarization” (Moon and Pyun, 1997; Muñoz et al., 2003; Gudić et al., 2005). The cathodic scan for the potentiodynamic polarization experiment was initiated at −1.7 V vs. Ag/AgCl which is different in comparison to CV and galvanostatic discharge studies, thus enabling the activation of Al. Consequently, as shown in the polarization curves (Figure 7), the corrosion potential of pure Al is located at more negative potentials. The activity of Al cannot be kept at anodic potentials due to the very low solubility and limited generation of OH^−^ leading to formation of passive oxide film.

The activation of Al on the Zn-10Al surface influences the electrochemical behavior of the alloy. According to the mixed potential theory of Wagner and Traud (Wagner and Traud, 1938), due to the electrochemical galvanic coupling reactions between the two metals in a binary alloy, the corrosion potential and corrosion current would be altered. The mixed potential theory suggests that the corrosion potential of an alloy should lie between the potentials of uncoupled two metals. When Al is activated, the potential of Zn-10Al alloy is found to be between Zn and Al potentials as shown in Figure 7. Moreover, the corrosion current density is also affected by alloying in line with the mixed potential theory.

### Discharge Behavior of the Alloy After Al Activation

In the potentiodynamic polarization studies, the effect of alloying was seen in terms of enhanced electrode potential with reduced corrosion current densities. For a battery application, however, the behavior of the electrode under galvanostatic polarization is of more interest. Therefore, different from the previous galvanostatic discharge experiments, an activation step was applied to enable the effect of alloying on the electrochemistry. Prior to discharge step, a cathodic potentiostatic pulse (−1.6 V vs. Ag/AgCl) was applied for 60 min.

**Figure 8:**
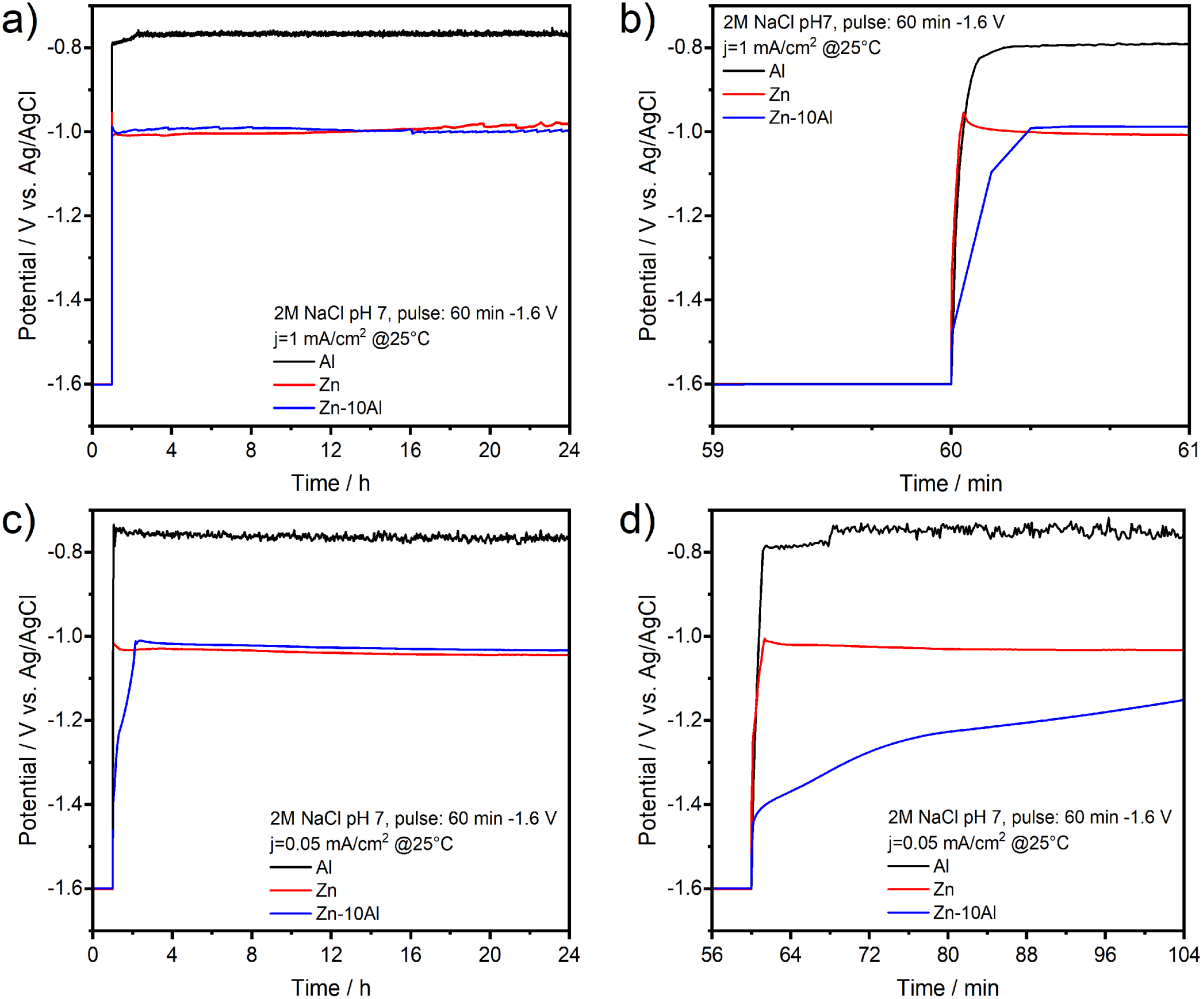
Galvanostatic discharge (stripping) experiments of pure Al and Zn, and Zn-10Al alloy electrodes: **(a)** Potential-time profile of electrodes under 1 mA/cm^2^ current density over 24 h, **(b)** The potential response upon initiation of the discharge with 1 mA/cm^2^, **(c)** Potential-time profile of electrodes under 0.05 mA/cm^2^ current density over 24 h, **(d)** The potential response upon initiation of the discharge with 0.05 mA/cm^2^. Initial 60 min of **(a,c)** correspond to the potentiostatic pulse of −1.6 V vs. Ag/AgCl which was applied to activate the Al constituents.

Figure 8 represents the potential-time profiles of the Al, Zn, and Zn-10Al alloy electrodes in 2M NaCl at pH 7 over 24 h. The first period of 60 min corresponds to the cathodic pulse. The discharge profiles of the Al, Zn, and Zn-10Al alloy electrodes are depicted in Figures 8a,b, where a current density of 1 mA/cm^2^ was applied directly after the cathodic pulse. Initiation of the galvanostatic discharge resulted in a sudden drop of the potential of Zn to −1.02 V vs. Ag/AgCl which is then stabilized around −1.00 V vs. Ag/AgCl. The potential of the Zn-10Al alloy first drops to −1.09 V vs. Ag/AgCl within few seconds, and then remains at very close to Zn potential for the rest of the time. The Al electrode also exhibits a rapid drop to −0.80 V vs. Ag/AgCl as soon as the current was applied. The potential plateau of Al was around −0.76 V vs. Ag/AgCl over 24 h.

The discharge profiles in Figures 8a,b show that imposing a 1 mA/cm^2^ discharge current density on the electrodes vanishes all the effects of activation step. After application of a cathodic pulse for 60 min, one might expect that the Al on the Zn-10Al alloy as well as pure Al electrodes should have been activated and thus, resulting in higher discharge potentials. On the contrary, the sudden drop of the electrode potentials to the values observed in Figure 4 suggests that the passive film was formed instantaneously on the Al compounds once the discharge was initiated. Most probably, the 1 mA/cm^2^ discharge current density was too high for the electrodes to sustain the active state. This can also be seen on the polarization curves (Figure 7), which illustrates that under 1 mA/cm^2^ current density the Al is already in the pitting stage.

**Figure 9:**
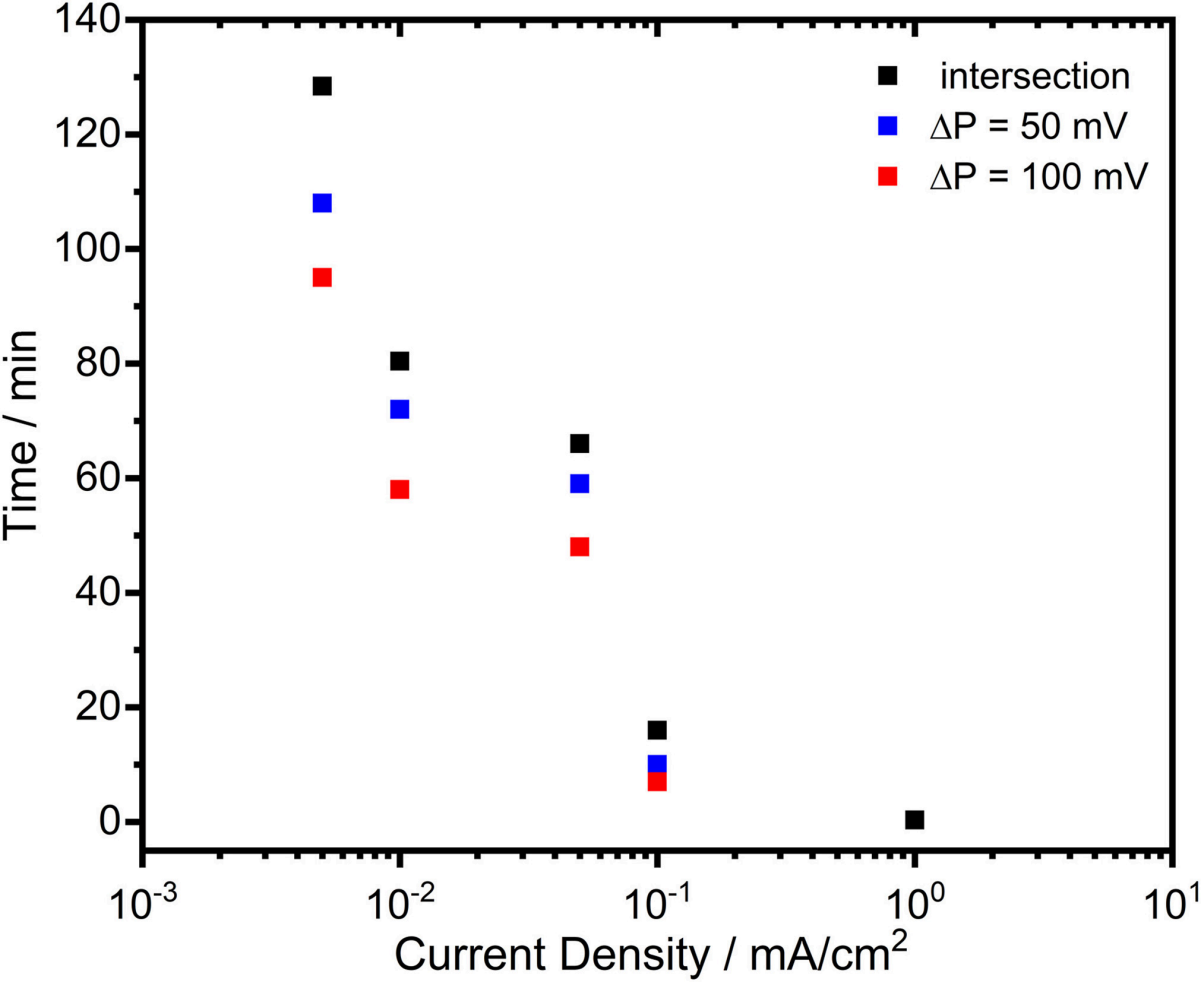
The time period analysis of the potential differences between Zn and Zn-10Al alloy electrodes during galvanostatic discharge experiments (see Figure 8) under various current densities.

In order to reveal the activation effect on the discharge profiles, significantly reduced current densities (0.005, 0.01, 0.05, 0.1 mA/cm^2^) were employed subsequent to the cathodic pulse. As an example, the potential-time profiles of the electrodes under 0.05 mA/cm^2^ are depicted in Figures 8c,d. By lowering the current density, there was no significant difference on the potential profiles of Zn and surprisingly of Al electrodes comparing to discharge profile with 1 mA/cm^2^. The discharge plateau of Zn was slightly shifted to more negative values (around −1.03 V vs. Ag/AgCl) while for the Al there was only few mV difference. A marked difference was observed for the potential profile of Zn-10Al alloy. Instead of a sudden drop, the potential decreased only gradually over time. Within the first few minutes of discharge, there was a potential difference of more than 400 mV between the Zn and alloy electrodes. Even after 50 min of anodic polarization, the potential was still in favor of the alloy by 100 mV. The effect of the enhanced negative potential of the alloy lasted for ~70 min after until the potential plateau of Zn was reached. A summary of the time period analysis of the potential differences between Zn and Zn-10Al alloy under various current densities is depicted in Figure 9.

Figure 9 clearly shows that reduced current densities (<0.1 mA/cm^2^) lead to significantly longer periods in which the discharge of Zn-10Al alloy can occur under stronger negative potentials than that of Zn. By discharging at 0.005 or 0.01 mA/cm^2^, enhanced potentials (by 100 mV) of alloy are maintained for almost 1 h. The re-establishment of the passive film on the surfaces takes place much more slowly in the alloy in comparison to pure Al which passivated almost immediately. The fact that the activation effect during discharge was observed only for the Zn-10Al alloy can be explained by the presence of Al and Zn constituents together on the surface. In the case of pure Al electrode, the compact passive oxide film is established immediately on the activated Al surface due to the very little solubility limits of the discharge products. Thereby, even under 0.05 mA/cm^2^, the surface passivates instantly and the discharge continues by the adsorption of Cl^−^ ions leading to initiation of pitting. For the alloy electrode, on the other hand, the Al is present with Zn in the alloy constituents which, most likely, prevents the formation of compact passive film on the Al. It is also known that Zn promotes the specific adsorption of Cl^−^ ions on it, which may retard the instant Al passivation (Baugh, 1979; Saidman and Bessone, 1997). Consequently, originating from the mixed potential theory, the alloy electrode exhibits a higher discharge potential until the Al compounds are partially exhausted or eventually passivated. Further investigations are required in order to understand the dissolution mechanism, whether it is a co-discharge of Zn and Al or only Al, during the enhanced potential periods.

Under different conditions, Zhang et al. (2012) investigated the selective dissolution of Zn and/or Al from a Zn-Al alloy by using online atomic emission spectroelectrochemistry (AESEC) method. Studies on the release rate of Zn and Al from Galvalume (55 wt.% Al, 43.4 wt.% Zn, 1.6 wt.% Si) in synthetic sea water (0.56M NaCl with pH 8.1) during ~40 min immersion and OCP experiments resulted in significantly higher zinc release rates in comparison to aluminum. Under an anodic potential of −400 mV vs. NHE, during the initial period mostly Zn was dissolved from the surface. Over time, the release rate of Zn was lowered gradually, while for Al it was increasing slowly before reaching a plateau. After 40 min of anodic polarization, 56% of the total amount of dissolved metals was aluminum under such high anodic potentials. Thereby, it was shown that co-dissolution of both metals during anodic polarization is possible.

All in all, the results obtained in this study clearly show that the electrochemical and corrosion behavior of zinc electrode can be influenced by alloying with aluminum under certain conditions. At low discharge currents, Al-alloying of the Zn provides enhanced discharge potentials for a limited time span after applying cathodic pulses to the cells. To be applicable as an anode material in a Zn–air battery, the stability of the discharge potentials with respect to discharge currents and time has to be considerably improved.

## Conclusion

Alloy electrodes containing 90 wt.% Zn and 10 wt.% Al were prepared to investigate their applicability as anodes in primary Zn–air batteries. The effect of Al addition to pure Zn electrodes was investigated with respect to discharge behavior, corrosion, local conductivities, and surface morphologies in 2M NaCl solutions at pH 7. Cyclic voltammograms and intermediate term discharge experiments (24 h) showed that the only active element in the alloy was Zn, while Al constituents remained inactive. Further investigations by laser scanning microscopy also illustrated that Zn rich phases were preferentially dissolved during discharge. Local conductivity measurements on the as-cast alloy surface by conductive atomic force microscopy confirmed that Al constituents were in the passive state. In order to activate Al constituents and reveal the effect of Al alloying on the electrochemistry, cathodic potentials were required which would generate OH^−^, and hence, hydrate/dissolve the aluminum oxide exposing bare Al surface due to local alkalization. Accordingly, potentiodynamic polarization curves revealed a clear shift on the potential of alloy toward more negative values due to mixed potential theory. Comparing to pure Zn, the corrosion current density and corrosion rate were also altered by alloying in favor of the Zn-10Al alloy. By fulfilling the precondition of Al activation prior to galvanostatic discharge experiments, the alloy provided temporarily enhanced discharge potentials over Zn electrode; however, the effect was limited to low current densities so far.

Overall, the effect of Al alloying was reported as temporarily enhanced discharge potential with lower corrosion rates in comparison to pure Zn electrode in neutral 2M NaCl electrolytes. Further investigations should be conducted in the direction of activating of Al possibly by using additives to electrolyte and/or electrode or changing the pH of the solution.

## Data Availability Statement

The datasets generated for this study are available on request to the corresponding author.

## Author Contributions

HK and YD: conceptualization. YD and SM: experimental work. YD: experimental data analysis and writing—original draft. HT, HK, FH, and R-AE: writing—review and editing. FH: AFM analysis. HT, HK, and R-AE: project administration. HK and R-AE: funding acquisition.

### Conflict of Interest

The authors declare that the research was conducted in the absence of any commercial or financial relationships that could be construed as a potential conflict of interest.
